# Defining a chromatin architecture that supports transcription at RNA polymerase II promoters

**DOI:** 10.1016/j.jbc.2024.107515

**Published:** 2024-06-28

**Authors:** Michael J. Fisher, Donal S. Luse

**Affiliations:** Department of Cardiovascular and Metabolic Sciences, Lerner Research Institute, Cleveland Clinic, Cleveland, Ohio, USA

**Keywords:** RNA polymerase II, promoter, nucleosome, TFIID, histone methylation

## Abstract

Mammalian RNA polymerase II preinitiation complexes assemble adjacent to a nucleosome whose proximal edge (NPE) is typically 40 to 50 bp downstream of the transcription start site. At active promoters, that +1 nucleosome is universally modified by trimethylation on lysine 4 of histone H3 (H3K4me3). The Pol II preinitiation complex only extends 35 bp beyond the transcription start site, but nucleosomal templates with an NPE at +51 are nearly inactive *in vitro* with promoters that lack a TATA element and thus depend on TFIID for promoter recognition. Significantly, this inhibition is relieved when the +1 nucleosome contains H3K4me3, which can interact with TFIID subunits. Here, we show that H3K4me3 templates with both TATA and TATA-less promoters are active with +35 NPEs when transcription is driven by TFIID. Templates with +20 NPE are also active but at reduced levels compared to +35 and +51 NPEs, consistent with a general inhibition of promoter function when the proximal nucleosome encroaches on the preinitiation complex. Remarkably, dinucleosome templates support transcription when H3K4me3 is only present in the distal nucleosome, suggesting that TFIID–H3K4me3 interaction does not require modification of the +1 nucleosome. Transcription reactions performed with an alternative protocol retaining most nuclear factors results primarily in early termination, with a minority of complexes successfully traversing the first nucleosome. In such reactions, the +1 nucleosome does not substantially affect the level of termination even with an NPE of +20, indicating that a nucleosome barrier is not a major driver of early termination by Pol II.

RNA polymerase II promoters *in vivo* have a stereotypical relationship with the surrounding chromatin. Active promoters in mammals reside in a nucleosome-depleted region extending from approximately 200 bp upstream to 40 to 50 bp downstream from the transcription start site (TSS) (1, 2). The proximal boundary of the first downstream (+1) nucleosome is typically located just beyond the expected footprint of the Pol II preinitiation complex (PIC) (1, 3). Close contacts of PICs and +1 nucleosomes have been demonstrated through immunoprecipitation of PICs from nuclease-treated nuclei, which reveals that a subset of the PIC-bearing fragments extend from the upstream PIC boundary to the downstream edge of typically-positioned +1 nucleosomes (4). The Pol II PIC extends roughly 35 bp downstream of the TSS for all Pol II promoters, regardless of the presence of a TATA element ∼30 bp upstream of the TSS (1, 4, 5). One might then imagine that the proximal edge of the +1 nucleosome (NPE) should not be closer than 35 bp downstream of the TSS at active promoters. A recent report nevertheless suggested that promoters with NPEs as short as +20 can support transcription *in vivo*, albeit at low levels (6). In that same study, *in vitro* transcription using a TATA promoter driven by TATA-binding protein (TBP) and purified general transcription factors demonstrated a low level of activity on nucleosomal templates with an NPE at +18. We recently tested nuclear extract–supported transcription of nucleosomal templates with various NPEs. TATA promoters were only weakly active compared to naked DNA controls even with NPEs of +51. However, when those reactions were supplemented with TBP, activity was recovered on the +51 NPE templates and significantly, +20 NPE templates supported about half the activity of the +51 NPE templates (7).

A major difficulty for extrapolating these *in vitro* experiments to the permissible nucleosome-TSS spacings *in vivo* is the nature of the Pol II promoter itself. The studies just cited all employed promoters with canonical TATA elements, which represent less than 5% of the promoters in mammalian genomes (8, 9). The assembly of PICs at TATA promoters can be driven by TBP alone, but in the absence of a TATA element, promoter recognition depends on the entire TFIID complex (5, 7, 10, 11, 12, 13, 14). At TATA-less promoters, TFIID primarily recognizes downstream promoter elements extending from roughly +18 to +35 (5, 15, 16). This would suggest that only TATA promoters could be active with NPEs closer to the TSS than about +35. Chen *et al.* (17) recently reported that TFIID can drive transcription on a nucleosomal template with an NPE of +41 but the promoter in that case contained a TATA element. We approached this question through *in vitro* transcription of two TATA-less promoters and a series of NPEs, from +51 to +100 (7). Reactions were performed with nuclear extracts so that recognition of the TATA-less promoters was driven by TFIID. The surprising result was that +51 NPE TATA-less templates supported almost no transcription; significant activity with these templates was only observed with NPEs greater than +70. Coupled with the observation noted above that +51 NPE TATA templates are active with TBP supplementation, our results are consistent with earlier studies that reported that chromatin templates support TBP-driven but not TFIID-driven transcription (18).

We imagined that restoring transcription on the TATA-less nucleosomal templates must require some additional component. A likely candidate was histone H3 trimethylated at lysine 4 (H3K4me3), since nucleosomes containing H3K4me3 are universally present near active Pol II promoters (19, 20). H3K4me3 has been shown to interact with the TAF3 subunit of TFIID (21, 22) and thus could activate TATA-less promoters through recruitment of TFIID. When we tested this directly, we found that the presence of H3K4me3 in the +1 nucleosome did restore activity of a TATA-less +51 NPE template (7). In addition, the presence of H3K4me3 substantially boosted the activity of TATA promoter templates with +51 NPEs when those reactions were driven by TFIID (7). Inactivation of TFIID from nuclear extracts eliminated transcription on the TATA-less promoters regardless of the presence of H3K4me3 in the nucleosomes (7). Activity could be restored with the depleted extracts by addition of free TBP but only on the TATA promoters.

The ability to study transcription driven by TFIID with immediately-proximal +1 nucleosomes allows us to determine whether promoters of either class, TATA-containing or TATA-less, can function on templates with NPEs ≤+35. We show here using H3K4me3-containing templates that both promoter types can support transcription, not only with +35 NPEs but at reduced levels with +20 NPEs. Thus, Pol II transcription complex assembly is possible even when the full TFIID footprint should be blocked by an adjacent nucleosome, apparently because TFIID can be recruited through interaction with H3K4me3. To extend our investigation of this point, we have constructed and transcribed dinucleosome templates on which either or both of the downstream nucleosomes can contain H3K4me3. As expected from the monosome templates, there is minimal TFIID-dependent transcription on these dinucleosome templates in the absence of H3K4me3, but activity is restored with the modified histones even when they are only present in the distal nucleosome. These results provide additional support for the idea that TFIID-dependent transcription in the presence of a promoter-proximal nucleosome requires an additional recruitment mechanism for TFIID.

Pol II complexes pause during the earliest stages of transcript elongation, typically at about 50 bp downstream of the TSS (1, 23, 24, 25). This is a significant control point for transcription since most of these complexes terminate with only a minority able to traverse the +1 nucleosome and advance to rapid productive elongation (26, 27, 28, 29, 30). The negative factors DSIF and NELF (31, 32, 33) slow early elongation which presumably renders Pol II complexes more susceptible to early termination by Pol II. The +1 nucleosome could also act to facilitate early termination, particularly since Pol II is liable to arrest during elongation on nucleosome templates (34, 35). When *in vitro* transcription reactions with nuclear extract are performed with minimal rinsing to retain nuclear factors, a majority of the Pol II early elongation complexes terminate. We report here that the presence of the +1 nucleosome in these reactions does not significantly affect the proportion of complexes that terminate, even with an NPE of +20. These results do not support a major role for the +1 nucleosome in facilitating termination during the earliest stages of transcript elongation by Pol II.

## Results

### TFIID-dependent transcription by Pol II can occur on nucleosomal templates with the proximal edge of the downstream nucleosome as close as +20

We showed earlier that TATA promoters are active on nucleosomal templates in a nuclear extract–based transcription system with an NPE at +20 but only when the reactions are supplemented with TBP (7). TFIID-driven transcription on the TATA templates is strongly inhibited by downstream nucleosomes without additional TBP, even with NPEs at +51, unless the nucleosome includes H3K4me3 (7). To identify the limits for close approach of the +1 nucleosome in TFIID-driven transcription, we constructed templates containing a native HNRNPAB (TATA-containing) core promoter linked to precisely positioned H3K4me3-containing mononucleosomes with NPEs at +20, +35, and +51. These templates were tested for transcriptional activity in a nuclear extract–based *in vitro* transcription assay and the results were compared to their corresponding naked DNA controls. The experimental approach is diagrammed in Fig. S1. All templates with positioning elements on which nucleosomes were to be assembled contained a unique BstX I restriction site within that element. Assembled templates were digested with BstX I before assays to eliminate any DNAs which did not contain nucleosomes.

**Figure 1:**
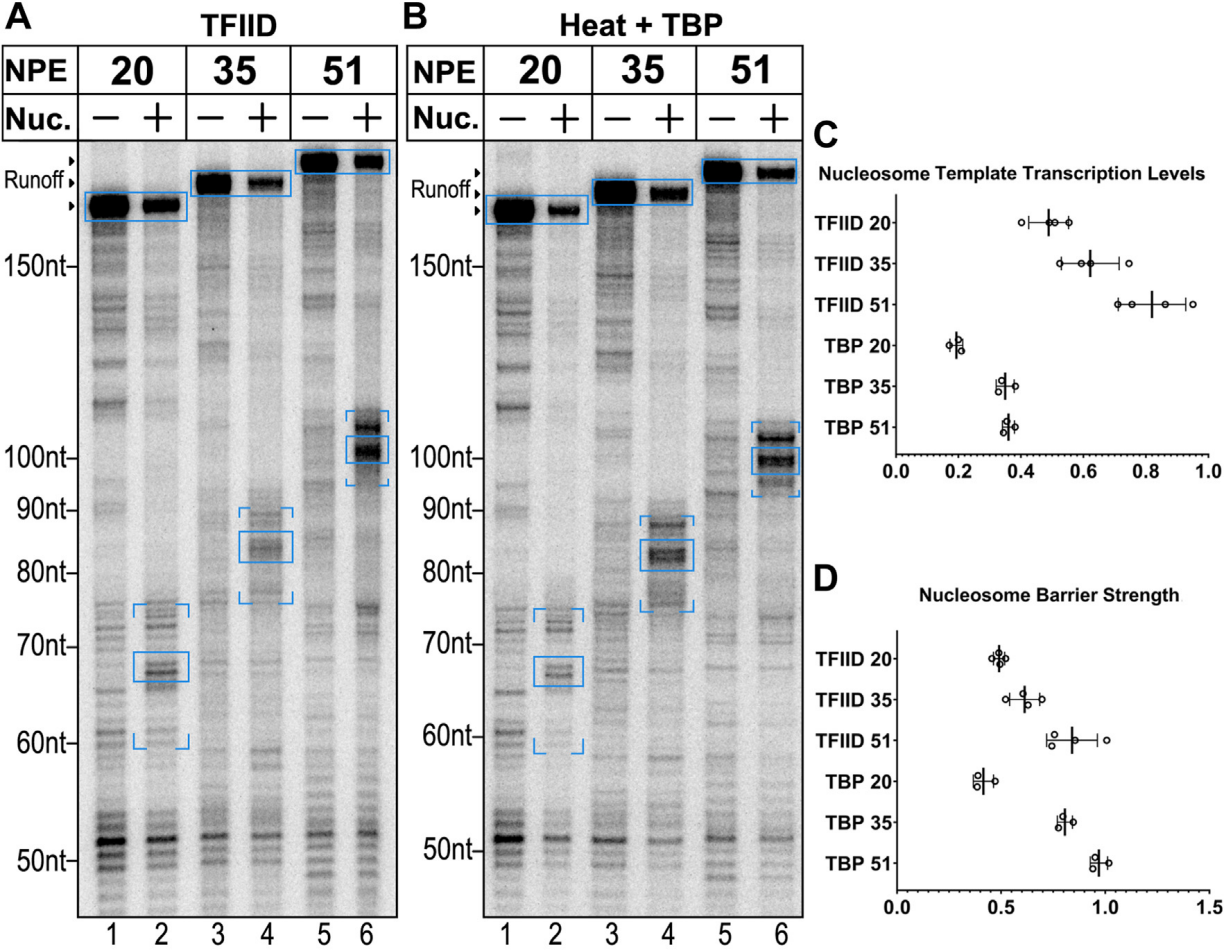
**H3K4me3 mononucleosome templates with HNRNPAB core promoters support transcription with NPEs 20 bp from the TSS.***A* and *B*, three templates with the HNRNPAB (TATA-containing) core promoter were transcribed as naked DNA or with an H3K4me3 nucleosome with NPEs at the indicated locations. Positions of run-off transcripts are indicated by the arrowheads to the *left* of each gel. Transcripts on the nucleosome templates that did not run off extended to a cluster of locations from 40 to 55 bp within the nucleosome, with the most prominent stops at 45 bp from the nucleosome edge. The three band clusters are indicated on the gel image with brackets below the 40 bp cluster and above the 55 bp cluster (See Fig. S4 for correlation of transcript lengths and corresponding locations of stop positions within the nucleosome.). The 45 bp barrier bands and the runoff bands are boxed and these were quantified for subsequent calculations. Experiments in *panels A* and *B* were carried out in an identical manner except *panel B* used nuclear extract heat treated for 15 min at 45 °C supplemented with 10 ng TBP per reaction (see Experimental procedures). *C*, nucleosome template transcription levels are calculated relative to their naked DNA counterparts as follows: (nucleosome runoff + nucleosome 45 barrier)/naked DNA runoff. Bars indicate the mean ± 1 SD. *D*, nucleosome barrier strength is the ratio of the 45 barrier signal to the runoff signal on nucleosome templates: nucleosome 45 barrier/nucleosome runoff. Bars indicate the mean ± 1 SD.

Earlier work consistently demonstrated that the major internal barrier to nucleosome traversal by Pol II is encountered at about 45 bp from the proximal nucleosome edge (7). To quantify our results, it was necessary to consider only those RNAs that unambiguously originated from correct initiation at our promoters. The cluster of bands at 45 bp within the nucleosome and the run-off both meet this test and thus we used the levels of these transcripts to determine the relative activity supported by our templates. The lengths of correctly initiated transcripts likely to be supported by each of these templates in relation to the position of the proximal nucleosome are diagrammed in Fig. S4. Transcription levels on the nucleosomal templates relative to the DNA control ranged from roughly 50 to 80%, decreasing as the NPE was moved closer to the TSS (Fig. 1*A* lanes 1–6, Fig. 1*C*). The lower level of activity on the NPE +35 templates relative to the NPE +51 templates likely reflects the fact that the proximal nucleosome boundary on the NPE +35 template is immediately adjacent to the expected downstream edge of the footprint of TFIID on that template (12, 36). It was surprising that the NPE +20 template supports any transcription since the nucleosome in this case should occupy about 15 bp at the downstream edge of TFIID’s DNA-binding footprint (12, 36). To isolate effects unrelated to TFIID, we heat-treated aliquots of nuclear extract to selectively inactivate TFIID (18, 37). With these extracts, transcription is completely dependent on added TBP (7) (Fig. S2), which allows us to compare transcription initiated with TFIID to transcription initiated with TBP alone. TBP-dependent transcription supported about half of the activity on the +20 NPE templates compared to the +51 NPE templates; unlike the case with TFIID-dependent transcription, activity on the +35 and +51 NPE templates was essentially the same (Fig. 1*B* lanes 1–6, Fig. 1*C*). This is consistent with the fact that TATA promoters are not necessarily dependent on promoter elements downstream of the TSS (9, 12, 38).

As expected from earlier studies (35, 39, 40), a substantial fraction of polymerases stopped during traversal of the nucleosome at a set of closely-spaced locations, the most prominent centered at 45 bp within the nucleosome (Fig. 1, *A* and *B*, lanes 2, 4 and 6). Unexpectedly, the relative proportion of pausing at this internal barrier *versus* full traversal of the nucleosome varied depending on the location of the +1 nucleosome. The ratio of transcripts stopped at the barrier to transcripts which advanced to template end increased the farther the nucleosome was located downstream of the TSS, regardless of whether transcription was driven by TFIID or by TBP alone (Fig. 1*D*). The dependence of barrier strength on location of the +1 nucleosome is considered further in the Discussion.

**Figure 2:**
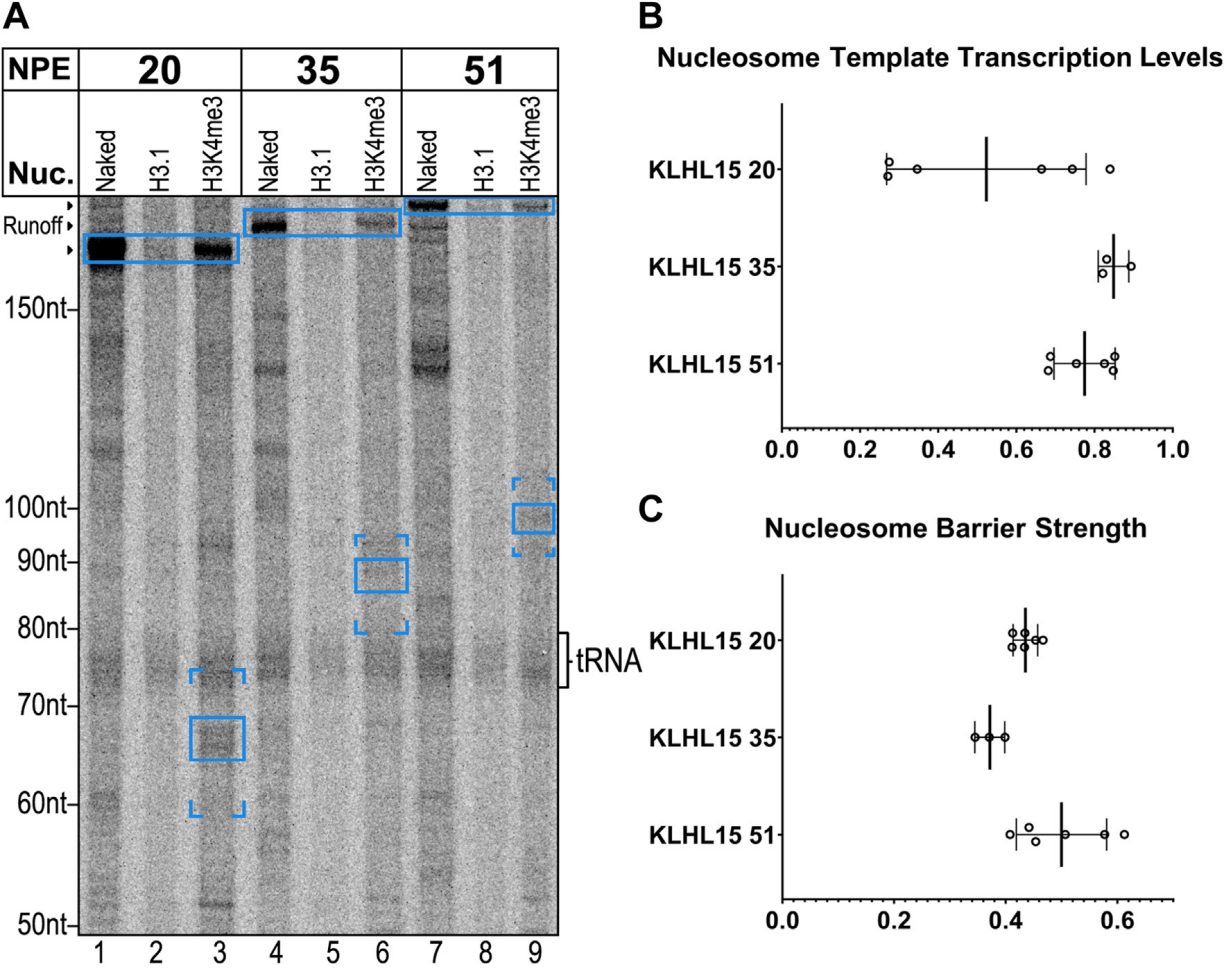
**TATA-less promoter KLHL15 supports transcription with H3K4me3 nucleosomes 20 bp from the TSS.***A*, three templates with the KLHL15 (TATA-less) core promoter were transcribed as naked DNA or with an H3.1 or H3K4me3 nucleosome with NPEs at the indicated locations. Positions of run-off transcripts are indicated by the arrowheads to the *left* of the gel. Nucleosome barriers are indicated on the gel image with brackets below the 40 bp cluster and above the 55 bp cluster. The major 45 bp barriers and the runoff bands are boxed and these were quantified for subsequent calculations. Contaminating labeled tRNA from the nuclear extract which could not be fully eliminated is indicated. *B*, nucleosome template transcription levels are calculated relative to their naked DNA counterparts as follows: (nucleosome runoff + nucleosome 45 barrier)/naked DNA runoff. Bars indicate the mean ± 1 SD. *C*, nucleosome barrier strength is the ratio of the 45 barrier signal to the runoff signal on nucleosome templates: nucleosome 45 barrier/nucleosome runoff. Bars indicate the mean ± 1 SD.

Since TATA promoters can rely on TFIIA/TBP–TATA interactions alone to drive PIC assembly (5, 38, 41), TFIID could recognize the TATA element through TBP regardless of downstream promoter elements (12). It is then possible that TFIID can still productively associate with TATA promoters through those upstream interactions even when downstream TFIID–DNA interactions are blocked. To test the importance of such downstream contacts on templates where they should be more important for promoter recognition (5, 10, 41), we constructed +20, +35, and +51 NPE templates containing a TATA-less core promoter, KLHL15. We have already demonstrated that this promoter is fully dependent on TFIID and inactive on +51 NPE templates that lack H3K4me3 (7). Our strategy for assembling templates by joining promoter and nucleosome-bearing segments through a common restriction site (Fig. S1) could result in the loss of some likely downstream promoter elements (DPEs). These elements, which encompass the downstream TFIID contacts, have been reported to extend from +18 to +35 (12, 16). The importance of retaining these sequences for the TATA-less KLHL15 promoter was emphasized by the results of our initial attempts to construct NPE +30 and +40 KLHL15 templates, where the downstream elements were partly or fully substituted with the nucleosome positioning sequences. As shown in Fig. S3, *A* and *B*, lanes 1 and 2, the promoter was inactivated by the complete loss of the downstream elements on the 30-DPE nucleosomal template even though the nucleosome contained H3K4me3. The KLHL15 +20 and +35 NPE templates were therefore designed to retain the native sequence downstream of +18 and minimize changes from the TSS to +18 (Table S1). When these templates were assayed after assembly with H3K4me3 nucleosomes, the +35 NPE KLHL15 template was nearly as active as the +51 NPE template and remarkably, the +20 NPE KLHL15 template retained roughly two-thirds of the +51 NPE activity (Fig. 2*A* lanes 1–9, Fig. 2*B*). The results in Figures 1*A* and 2*A* together emphasize the profound role for H3K4me3 in not only reversing the inhibition of TFIID by promoter-proximal nucleosomes but also in actively facilitating productive association of TFIID with the promoter even when many of the downstream TFIID contacts are occluded. Unlike the case with the TATA promoter, there was no correlation between the distance from the TSS to the NPE and extent of nucleosome traversal (Fig. 2*C*).

### Nucleosome attenuation of transcription extends beyond the +1 nucleosome

Nucleosomes with the H3K4me3 modification are always present near active Pol II promoters (19, 22), but the modification is not limited to the +1 nucleosome as modeled in our mononucleosome template design. To explore potential roles for H3K4me3 beyond the +1 nucleosome, we developed a template system containing two precisely positioned downstream nucleosomes spaced 50 bp apart. Transcription on these templates is driven by the adenovirus major late (AML) TATA-containing promoter instead of HNRNPAB as in Figure 1. We showed earlier (7) that these TATA promoters behave similarly with respect to the effect of proximal nucleosomes, ± H3K4me3. We chose AML for the dinucleosome templates because we had noticed that the HNRNPAB promoter also supports some antisense transcription, probably by recognition of the TATA element in the opposite orientation. The cluster of 50 nt long RNAs in the Figure 1 reactions arise from this antisense transcription. Use of the AML promoter allowed us to avoid these complications.

**Figure 3:**
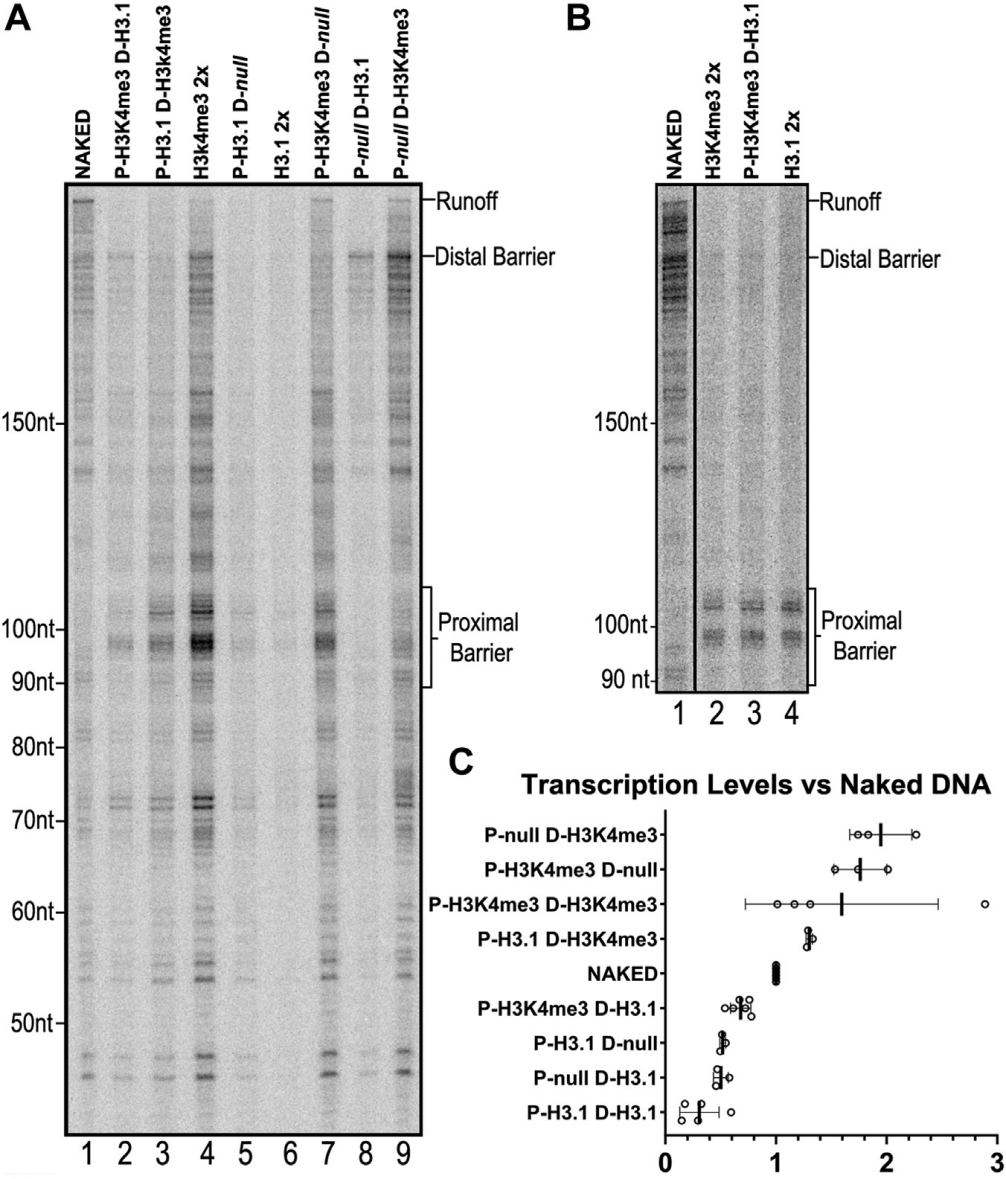
**Position and identity of nucleosomes in tandem influence transcription levels.***A*, DNA templates with the AML core promoter and two nucleosome positioning elements downstream were transcribed with the indicated combinations of nucleosomes assembled at the proximal “P” or distal “D” locations. For templates where one positioning element was not assembled, that location is labeled *null*. Templates with identical nucleosomes on both elements are labeled “2x”. Runoffs, proximal nucleosomal barriers for lanes 2 to 7, and distal nucleosomal barriers for lanes 2 to 4, 6, 8, and 9 are indicated. *B*, the experiment in *panel A* was repeated with representative templates using nuclear extract heat-treated for 15 min at 45 °C supplemented with 10 ng TBP per reaction. *C*, background-corrected levels of transcription for each template shown in *panel A* are quantified by measuring the total signal in each lane shown and compared as a ratio to the signal for naked DNA templates. Templates are ranked from highest to lowest by average transcription levels. Bars indicate the mean ± 1 SD.

As diagramed in Fig. S1, the two nucleosomes are assembled separately before being joined. By using this approach, we can generate templates with one or two downstream nucleosomes, one or both of which can contain H3K4me3 or histone H3.1. In all cases, the proximal edge of a nucleosome was no closer to the TSS than +51. We tested these templates in the nuclear extract–based transcription assay as performed with the mononucleosome templates. As anticipated (Fig. 3*A*, lanes 5, 6 and 8; Fig. 3*C*), templates with only unmodified H3.1 nucleosomes supported much less transcription overall as compared to the naked DNA controls. This was true even for templates with a single H3.1 nucleosome in the distal position (P-null, D-H3.1; Fig. 3*A* lane 8) where the NPE is +248. Activity increased when H3K4me3 nucleosomes were present but the relationship of modified and nonmodified nucleosome positions to transcriptional activity was unexpected. It is especially noteworthy that H3K4me3 was stimulatory in the distal position, regardless of whether the proximal position contained an H3.1 nucleosome (lane 3) or no nucleosome (lane 9). For the templates which contained both an H3.1 and an H3K4me3 nucleosome, the presence of H3.1 was inhibitory: activity was lower on the P-H3K4me3, D-H3.1 template relative to P-H3K4me3, D-null (compare lanes 2 and 7), and similarly lower on the P-H3.1, D-H3K4me3 template relative to P-null, D-H3H4me3 (compare lanes 3 and 9).

The P-null D-H3K4me3 and P-H3K4me3 D-null templates support as much as twice the activity compared to the naked DNA controls (Fig. 3*C*), in contrast to the NPE +51 H3K4me3 monosome templates in Figure 1 which supported less activity relative to the controls (Fig. 1*C*). The templates used in Figure 3 all contain much longer DNAs than those in Figures 1 and 2. We suggest that the unexpected greater activity seen in Figure 3 reflects an enhanced ability to recruit TFIID on the longer templates. Whatever the mechanistic basis, the results in Figure 3 demonstrate the competitive effect of H3.1 nucleosomes *versus* the activating effect of the H3K4me3 nucleosomes. As an essential control, selected templates containing proximal and distal nucleosomes were assayed with heat-treated extracts where transcription depends on added TBP. In those reactions, transcription activity was the same on all three templates, emphasizing that both the inhibitory and activating effects of the nucleosomes function through TFIID (Fig. 3*B*, lanes 2–4).

### Termination during the early stages of transcript elongation *in vitro* is not affected by an immediately proximal nucleosome

During the earliest stages of transcript elongation *in vivo*, polymerases pause at roughly the point where they encounter the +1 nucleosome (1, 3). Importantly, only a fraction of these complexes advance into productive elongation while most terminate transcription (26, 27, 28, 29, 30). Given the barrier to transcript elongation presented by the +1 nucleosome, it was important to determine whether that barrier actually influences the partitioning between productive elongation and termination. To investigate this, we needed to establish that our extract-based transcription system does support the generation of both efficient elongation complexes and termination early in elongation, as suggested by earlier studies (42). The protocol we use for the nuclear extract–supported transcriptions as in Figure 1, Figure 2, Figure 3 involves a rinsing step of the initial pulse-labeled bead-bound reactions. This is necessary to remove the nontranscriptionally labeled RNAs, particularly tRNAs, but factors that promote elongation or drive termination should also be lost. To test for the possibility that these activities can be observed in our extract-based reactions, we modified our approach and directly chased the initial pulse reactions with excess nonlabeled NTPs (no-initial-rinse protocol, Fig. S1). After the chase, reactions were divided into template-engaged and released fractions and the bead-attached templates were rinsed to remove labeled tRNAs. To separate transcripts from nontemplated RNAs in the released fraction, transcripts were specifically recovered by hybridization to oligonucleotides complementary to the initial 20 nt of the transcripts.

**Figure 4:**
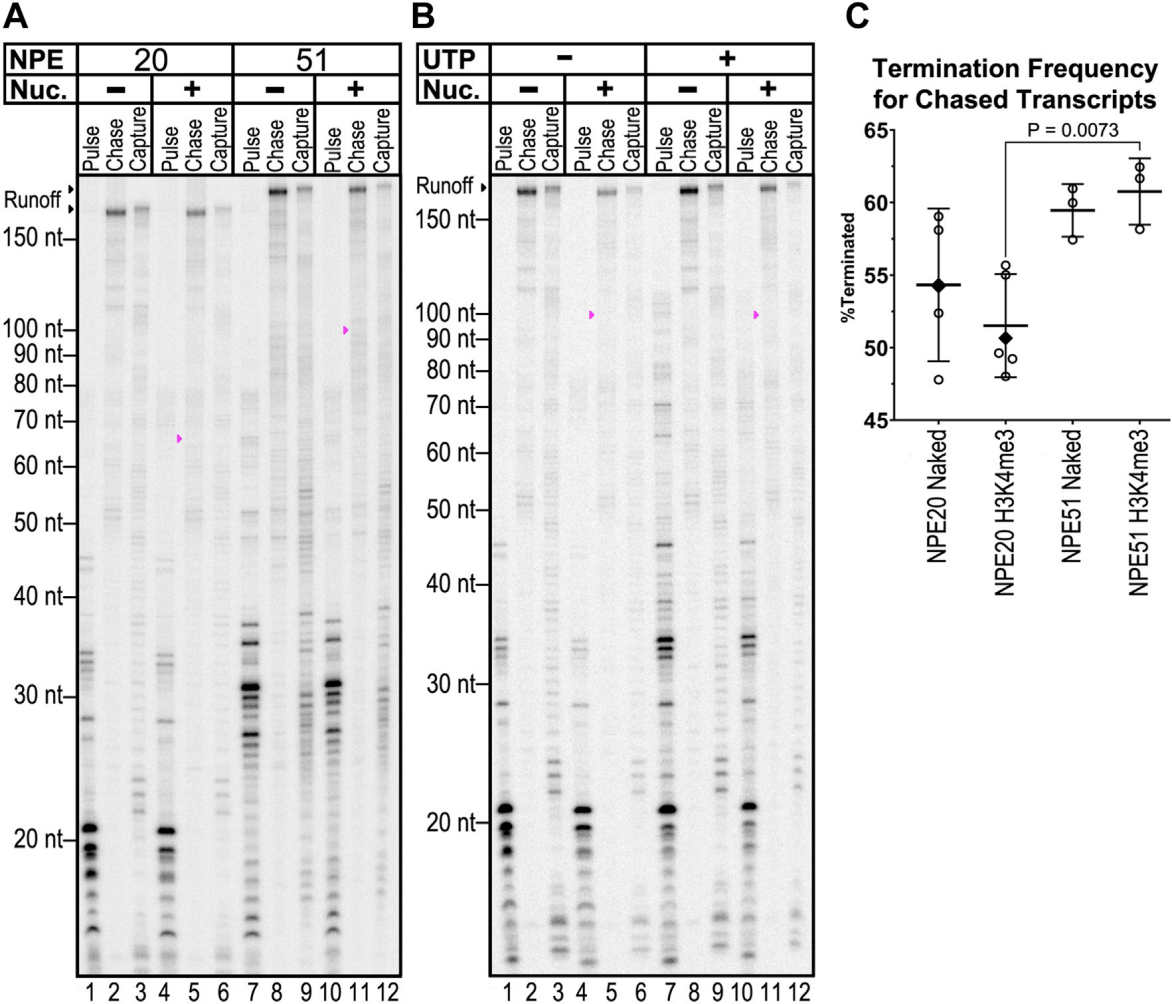
**Chasing pulse-labeled reactions before rinsing reveals effective nucleosome traversal by template-engaged complexes and early termination for a majority of complexes.***A*, templates with HNRNPAB core promoters were transcribed as either H3K4me3 mononucleosome templates with NPEs indicated or as naked DNA. The transcription assay was modified to take a sample after the pulse (Pulse), retain the nuclear extract during the chase, and then separate engaged (Chase) from released (Capture) transcripts (See Fig. S1). Runoff transcripts are indicated by the *black* arrowheads to the *left* of the gel. *Magenta arrowheads* on lanes 5 and 11 indicate the expected positions for transcripts corresponding to stops at the major barrier 45 bp within the nucleosome. *B*, HNRNPAB NPE 20 templates were transcribed as in panel A ± 1 μM additional UTP in the pulse mixture. Runoff transcripts are indicated by the *black* arrowhead to the *left* of the gel. *Magenta* arrowheads on lanes 5 and 11 indicate the expected positions for transcripts corresponding to stops at the major barrier 45 bp within the nucleosome. *C*, termination frequency for chased transcripts was calculated as a percentage of the sum of background-corrected signals from 10 to 60 bp, 100 bp – runoff signal in the “capture” lane, and 100 bp – runoff signal in the “chase” lane: [“10–60 bp capture”/(“10–60 bp capture” + “100 bp to runoff capture” + “100 bp to runoff chase”)] x 100%. Bars indicate the mean ± 1 SD. Terminated transcript frequency for UTP-supplemented pulses is indicated with *black* diamonds.

Nucleosomal templates with +51 NPEs and HNRNPAB promoters were transcribed along with corresponding naked DNA controls using the no-initial-rinse protocol. The resulting pulse-labeled RNAs and both the template-retained (chase) and released (capture) RNAs are shown in lanes 7 to 12 of Figure 4*A*. On the control reactions with naked DNA, the retained transcripts (lane 8) primarily extend to nearly the end of the template, probably prevented from completely running off by biotin-conjugated streptavidin that anchors the DNA on the beads. The corresponding reactions on the +51 NPE nucleosomal templates show essentially the same pattern of retained RNAs (lane 11). Importantly, there was no evidence of pausing centered at 45 bp within the nucleosome (magenta arrowhead), in contrast to reactions rinsed before chase as in Figure 1. The transcripts released through termination on the naked DNA +51 NPE templates were heterogeneous but mostly <60 nt (lane 9). The released fraction also contained RNAs slightly longer than the longest template-retained transcripts; these were presumably generated by polymerases that did not terminate but transcribed fully to template end. The length distribution of the released RNAs from the +51 NPE nucleosomal templates (lane 12) was similar to the distribution seen with the naked DNA reactions up to roughly the proximal edge of the nucleosome, with relatively fewer transcripts downstream of that point. A comparison of the amount of RNA recovered in the template-engaged *versus* the terminated fractions on the NPE +51 templates shows that the majority terminated (Fig. 4*C*). Crucially, this distribution does not change between nucleosomal and naked DNA templates (Fig. 4*C*). This partitioning of early elongation complexes into elongation-competent and terminated roughly mirrors the expected relative fates for promoter-proximal transcription complexes *in vivo* (26, 28).

Since termination in our system mostly occurs before Pol II would encounter a proximal nucleosome edge at +51, we considered the possibility that imposing the nucleosomal barrier much earlier in transcription could more effectively reveal nucleosome-specific effects. We took advantage of our observation that the HNRNPAB promoter supports transcription on NPE +20 templates (Fig. 1). As shown in lanes 1 to 6 of Figure 4*A*, the naked DNA and nucleosomal no-initial-rinse reactions on these NPE +20 templates gave indistinguishable results regarding the relative levels of template-engaged *versus* released RNAs. This is not consistent with the idea that immediately imposing a nucleosomal barrier to early elongation would make complexes more likely to terminate. Instead, a significantly smaller proportion of complexes terminated on the NPE +20 templates (*p* = 0.0073) compared to the NPE +51 templates. The fact that a +20 NPE nucleosome does not affect the productive elongation/termination partition extends our understanding of the role of local chromatin structure on Pol II promoter function and thus more fully defines the functional role of the local chromatin structure in transcription.

The difference in termination levels for the NPE +20 and NPE +51 templates should be interpreted cautiously, since the sequence traversed by early elongation complexes on the two templates must by design be different. As an initial test of the importance of pulse conditions on the same template, we repeated the no-initial-rinse reactions on the NPE +20 template, but one set of reactions was allowed to progress farther downstream in the pulse step because of additional UTP (Fig. 4*B*). Complexes with UTP added did generate some longer RNAs in the pulse step, out to about 50 nt (compare lanes 1 and 4 with lanes 7 and 10). However, these differences did not affect the level of termination in the resulting chase reactions on either the naked DNA or nucleosomal templates (Fig. 4, *B* and *C*).

## Discussion

### TFIID competes for DNA with nucleosomes close to the TSS of active promoters

Assembly of the Pol II PIC begins with recognition of the promoter by TFIID (1, 5, 14). TFIID protects sequences on naked promoter DNA from roughly 40 bp upstream of the TSS to 35 bp downstream (12). In the absence of an upstream TATA sequence, downstream elements extending from +18 to +35 are essential for promoter function (10, 11, 13). One might imagine that promoter activity would be incompatible with nucleosome proximal edges closer than 35 bp downstream of the TSS. However, we previously established that promoter-adjacent nucleosomes, even those positioned well downstream of +35, are powerful inhibitors of TFIID-driven transcription *in vitro* (7). PIC assembly in the presence of a promoter-proximal nucleosome apparently requires some additional component, an activator, that recruits TFIID to the promoter region. In this study, the H3K4me3 modification serves that role. We show here that this recruitment is so effective that a TATA-less promoter is nevertheless active with an H3K4me3 nucleosome whose proximal edge is +20, which should occlude important downstream elements. Thus, we are defining the permissive chromatin architecture for promoters: the proximal nucleosome is more inhibitory to transcription than expected when TFIID is not recruited, but less inhibitory than expected when TFIID is recruited. Importantly, we also show here that the activating modification need not be present in the +1 nucleosome.

While the presence of the modification is necessary for activity on TATA-less promoters, it is not sufficient. Substituting the DPE of KLHL15 with an arbitrary sequence (Table S1) reduces promoter strength on naked DNA and ablates activity on nucleosomal templates with a +30 NPE even if that nucleosome has the H3K4me3 modification (Fig. S3). It was suggested that TFIID can adopt a rearranged conformation, allowing the complex to bind DNA primarily through association of TFIIA/TBP (5, 12) in an “upstream first” mode of association. This scenario would account for the TFIID-driven activity we observed on the TATA promoter NPE +20 template, which lacks the native downstream promoter elements (Fig. 1*A* lanes 1,2). That model should not apply for TATA-less promoters like KLHL15. We have previously shown that TBP supplementation does not restore activity for KLHL15 promoters on NPE +51 templates with unmodified nucleosomes (7). Additionally, earlier studies demonstrated that mutating or deleting downstream promoter elements reduces promoter strength on other TATA-less promoters (11, 13). The transcriptional activity of the KLHL15 NPE +20 template thus indicates that TFIID can successfully compete with an occluding nucleosome for its downstream DNA contacts. Earlier work showed that DNA on the nucleosome surface associated with the H2A-H2B dimers can transiently dissociate over time scales that are relevant to our 30 min assembly for Pol II PICs. Incubation of 147 bp nucleosome core particles with exonuclease III showed that the initial 10 to 20 bp of nucleosomal DNA are accessible to the exonuclease (43). Similar tests using restriction enzymes demonstrated that the ends of the DNA wrapped on a nucleosome-positioning sequence can be available for cleavage (44). Such dissociation on the KLHL15 NPE +20 template would allow TFIID to productively associate with the DPE and initiate PIC assembly.

On all templates, XPB translocates downstream DNA into the PIC to drive promoter melting (45, 46); see also (6). Once the nascent RNA is 3 nt long, 16 bp of template DNA have already been translocated upstream (46). Further translocation occurs until promoter clearance is achieved after a ∼9 nt transcript has been made (46, 47). Thus, on templates with an NPE at +20 or +35, roughly 20 bp of DNA will be displaced from the proximal face of the +1 nucleosome before Pol II is fully committed to elongation. On +20 NPE templates, PIC assembly itself could displace as much as 15 bp of DNA from the nucleosome before XPB acts. These effects together should result in Pol II advancing over unbound DNA well into the 601 positioning element and thereby reduce the difficulty for Pol II in crossing the major nucleosome barrier to elongation immediately downstream. This would explain the increased readthrough frequency as the nucleosome is located closer to the TSS, as seen in Figure 1*D*. Traversal is high on both the KLHL15 +20 NPE template and the HNRNPAB +20 NPE template when transcription is driven by TFIID, but traversal is lower for the HNRNPAB +35 NPE template than the KLHL15 +35 NPE template (Figs. 1*D* and 2*C*). This is consistent with TATA-containing promoters not being fully dependent on DPE contacts, even when transcription is driven by TFIID. Thus, initial unwrapping of the nucleosome to accommodate PIC formation may not be as extensive at the HNRNPAB promoter compared to KLHL15, giving less support for elongation through the major nucleosomal barrier.

### Chromatin-directed transcription regulation is likely a function of the three dimensional arrangement of nucleosomes and the promoter

**Figure 5:**
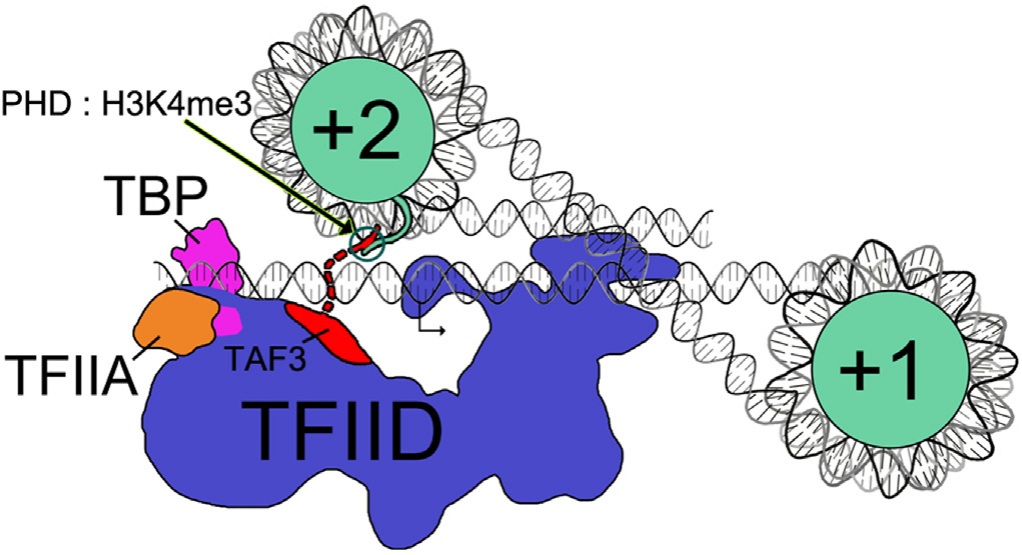
**A graphical representation of dinucleosome templates.** This model depicts a dinucleosome template with nucleosomes assembled on both positioning elements, extending from just upstream of the TATA sequence to the end of the template. Relative sizes and positions of the proteins and DNA are based on known structural data for each and are depicted approximately to scale. DNA is assumed to be straight outside of the protein complexes. The TSS is indicated with an arrow. Histones are represented in *teal*, the template strand in *silver*, and the nontemplate strand in black. TFIID is shown in an initial TBP-loading state based on cryo EM structure 7EGD (5). The section of TAF3 within TFIID whose location was resolved in that structure is shown in *red*. This includes only the 127 N-terminal amino acids. The PHD domain of TAF3 is located at the far C-terminal end of the 929 residue protein; the structure of the extension toward the nucleosome is not known and thus is simply modeled in the diagram with a *dashed* line. The *circle* designates the expected interaction between the TAF3 PHD domain and trimethylated lysine 4 of histone H3. Given that the ultimate alignment of TFIID on promoter DNA within the PIC is essentially the same relative to the TSS for both TATA and TATA-less promoters (5), this model should apply to promoters of both classes.

Several recent reports have suggested that the +1 nucleosome is involved in the recruitment of TFIID to promoters (15, 17). However, the results in Figure 3 show that for certain configurations of the dinucleosome templates, a +2 nucleosome can have a greater effect on transcription than the +1, depending on its modification status. Figure 5 shows a model for these dinucleosome templates incorporating the known dimensions of the nucleosome, the 50 bp spacer between the nucleosomes, and the DNA-binding region of TFIID. The quantitative effects of modified and nonmodified nucleosomes on the dinucleosome templates are best explained by considering the relative locations of the nucleosomes in three dimensions as diagrammed in the Figure. The model predicts that the +2 nucleosome on these templates is closer to TFIID at the promoter than the +1 nucleosome, allowing greater regulatory access for the flexible H3K4me3 tail to the PHD domain of TAF3 in the lobe A of TFIID. The ability of H3K4me3 to activate transcription from the +2 nucleosome even though the +1 nucleosome contains H3.1 is consistent with this model (Fig. 3*A*, lane 3). The results with the dinucleosome templates reinforce the point from our original study (7) that the presence of an unmodified nucleosome is inhibitory wherever it is located on the DNA, even with an NPE of +248 (Fig. 3*A*, lane 8). The inhibitory effect of H3.1 nucleosomes is only seen when transcription is driven with TFIID (Fig. 3*B*). Thus, a plausible explanation is that unmodified nucleosomes interact with TFIID to prevent its productive interaction with promoters. On hybrid templates with both unmodified and H3K4me3 nucleosomes, we can view the resulting level of transcriptional activity as reflecting a competition between these activating and inhibitory effects. The Figure 5 model is useful in interpreting the results with the hybrid templates. On the P-H3K4me3, D-3.1 template, the inhibitory unmodified nucleosome is predicted to be closer to promoter-bound TFIID than the H3K4me3 nucleosome, while the activating interaction is predicted to be closer to TFIID on the P-3.1, D-H3K4me3. This is consistent with the greater transcriptional activity of the P-3.1, D-H3K4me3 template (Fig. 3*A* lane 3) than the P-H3K4me3, D-3.1 template (Fig. 3*A*, lane 2). Both the unmodified and the H3K4me3 nucleosomes are effective as inhibitors or activators of transcription when they are present as the sole nucleosomes in the +2 position (Fig. 3*A*, P-null, D-3.1, lane 8 and P-null, D-H3K4me3, lane 9). On those templates, each nucleosome is ∼250 bp from the TFIID-binding site; at that distance, DNA is sufficiently flexible to allow the nucleosomes to transiently associate with TFIID (48).

### Early elongation complexes terminate to different extents regardless of the presence of a proximal nucleosome

In the *in vitro* transcription assays used for Figure 1, Figure 2, Figure 3, factors from the nuclear extract were removed from the bead-attached templates in the washing step after PIC assembly. When those complexes were chased, polymerases could not efficiently traverse the major barrier ∼45 bp within the nucleosome, at least for NPEs at +51 and beyond (7). As shown in Figure 4, when nuclear factors are retained in the chase, polymerases that remain on the template elongate efficiently and cross the nucleosomal barrier (lanes 5 and 11, panels A and B). A larger fraction of the complexes on the nucleosomal templates terminate transcription, releasing RNAs which are primarily 50 nt or less (lanes 6 and 12, panels A and B). In the naked DNA reactions in Figure 4, more complexes terminated on the NPE +51 template compared to the NPE +20 template (Fig. 4*C*). The negative factors DSIF and NELF cannot load until the nascent RNA has extended beyond 18 nt (49) and longer RNAs bind these factors more effectively (50). DSIF and NELF exclude the binding of the positive component PAF1 until DSIF is phosphorylated by P-TEFb and NELF is released (51, 52). The Integrator complex, which is a major driver of termination (53), binds to DSIF/NELF-containing complexes (54). Thus, differences in the loading of DSIF/NELF on the nascent RNA are likely to be important in the relative ability of elongation and termination factors to control the fate of Pol II complexes immediately downstream of promoter clearance. In this context, it is important to note that Pol II complexes are prone to backtracking when stopped early in elongation as far downstream as +27 to +32 (55). Backtracking for these complexes is extensive (>10 nt, depending on the location at which polymerase halted), which will withdraw the upstream RNA into the polymerase and conceal potential sites for factor binding. The differences in termination levels on the naked NPE +20 *versus* NPE +51 templates could be based in part on the backtracking status of the complexes, as well as the different RNA sequences in the nascent RNAs. The presence of an immediately downstream nucleosome could affect the relative access of factors and therefore the balance between termination and effective elongation but that was not the case, at least for the two examples we tested. As noted above, particularly for complexes on the +20 NPE template, much of the DNA originally bound to the proximal H2A-H2B dimer should already be unwrapped as polymerase enters elongation. If loading of elongation or termination factors is rapid relative to the initial elongation rate, on templates with immediately proximal nucleosomes, the fate of complexes may be determined before the major nucleosomal barrier is encountered.

## Experimental procedures

### DNA sequences

Promoter DNA fragments for AML, HNRNPAB, and KLHL15 were amplified from plasmids containing the cloned sequences described previously (7). The distance from the TSS to the NPE of promoter DNA fragments were determined by the downstream primers used. The upstream primer for AML was M13R and the downstream primer was AML51_AvaI (IDT; Sequences shown in Table S1). The upstream primer for HNRNPAB was HNRNPAB-96 and the downstream primers were HNRNP51_AvaI, HNRNP35_AvaI, and HNRNP20_AvaI (IDT; Sequences shown in Table S1). The upstream primer for KLHL15 was M13F and the downstream primers were KLHL15-51_AvaI, KLHL15-35_AvaI, and KLHL15-20_AvaI (IDT; Sequences shown in Table S1).

DNA fragments containing a nucleosome positioning sequence (NPS) were amplified from plasmids as described previously (7). DNA fragments used for assembly in the tandem array template required a different set of primers and for NPSs which were to remain naked in the final template, a plasmid containing the modified 601 element lacking the BstX I site (601S, shown in Table S1). DNA fragments containing an NPS to be used to assemble the KLHL15-20 and KLHL15-35 templates required their own unique upstream primers for amplification: 601KLHL15-20_AvaI and 601KLHL15-35_AvaI, respectively (IDT; shown in Table S1). DNA primers (IDT; shown in Table S1) 601R_AvaI and 601S_Rev_AvaII were used to amplify the proximal NPS DNA, and 601S_Fwd_AvaII and 601R_Rev_Biot were used to amplify the distal NPS DNA.

### DNA template preparation

Nucleosomes were reconstituted by a salt dilution procedure as previously described (7). Histone dimers and octamers were purchased from ActiveMotif. Tetramers containing H3K4me3 and H4 histones were custom ordered from EpiCypher. DNA template fragment preparations and template assemblies were performed as previously described (7), modified in the following ways. The tandem array template proximal NPS fragment was double-digested with 10 U of Ava I and 10 U of Ava II, and the distal NPS fragment was digested with 10 U of Ava II instead of Ava I. Tandem array template assemblies used 50 ng each of reconstituted proximal and distal nucleosome fragments, 20 μl 10x T4 DNA ligase buffer, 800 U T4 DNA ligase (NEB) in a final volume of 200 μl. If the template lacked either a proximal or a distal nucleosome, the NPS used for the empty spot lacked a BstX I site. Following conjugation to ferromagnetic beads, all nucleosome-bearing templates were digested with BstX I as previously described (7).

### Transcription assays

*In vitro* transcription reactions were performed as previously described (7). In summary: PICs were formed by incubating DNA templates in transcription buffer containing HeLa nuclear extract. Reactions were initiated with limiting NTPs with radioactive CTP, washed, then chased with excess NTPs. Transcripts were analyzed by urea PAGE and exposed to phosphorimager screens to produce an autoradiogram. The following modifications were made to the original procedure: for the experiments that were supplemented with TBP, reactions were supplemented with an additional 1 μl of 10 ng/μl TBP in BC100 buffer before incubating to form a PIC. For tandem array experiments, an 8% acrylamide denaturing PAGE was used instead of 10% and the gel was run 90 min at 54 W, holding power constant. For the nuclear extract retention and RNA capture experiments, reactions were initiated by adding 3.5 μl limiting NTPs as described previously (7), the reactions were then divided equally into two separate tubes, 14 μl each, and incubated for 150 s at 30 °C. One reaction was stopped while the other reaction was chased. If the reactions were chased, then 1.5 μl of 2 mM each of ATP, CTP, GTP, and UTP was added and the reaction was incubated for another 300 s at 30 °C. After chasing, the magnetic beads were pelleted, the supernate was transferred to a separate test tube containing 35 μl stop solution (20 mM EDTA, 200 mM NaCl, 1% SDS, 100 μg/ml Proteinase K, 20 μg/ml tRNA), mixed, and incubated at 30 °C for at least 10 min. After removing the supernate, the reaction on the beads was stopped. The stop procedure is as follows: pelleted beads were washed twice with 50 μl XM75 supplemented with 0.002% Igepal CA-630, resuspended in 15 μl XM75, then stopped by adding 35 μl stop solution and incubated at 30 °C for 5 min. After the stopped reactions were incubated, they were phenol:chloroform:isoamyl alcohol extracted as described previously (7). Only phenol:chloroform:isoamyl alcohol–extracted reactions from pelleted bead samples were ethanol precipitated and resuspended in loading buffer as previously described (7). Chase supernate samples were instead captured by adding 25 μl high salt buffer (500 mM NaCl, 30 mM Tris pH 7.5) and 4 μl 200 nM compatible capture oligonucleotide. Capture oligonucleotides were 3′ biotinylated DNA oligonucleotides (IDT) reverse-complementary to the 5′ ends of the predicted transcripts for the respective templates (C_HNRNPAB20 for the NPE 20 template, C_HNRNPAB51 for the NPE 51 template, shown in Table S1). The oligos were hybridized to the transcripts by incubating for 16 h at 24 °C, then conjugated to Dynabeads M-280 streptavidin (Invitrogen). The conjugated beads were washed twice with 75 μl capture wash buffer (10 mM Tris–Cl pH 7.5, 5 mM EDTA, 50 mM NaCl, 50 μg/ml tRNA), then resuspended in 7 M urea sequencing gel-loading buffer. Samples were analyzed by denaturing PAGE as previously described (7).

### Image analysis

Images were analyzed with ImageQuant TL 1D gel analysis software. Signals were quantified by first using stepwise manual lane creation function to draw lane-bounding boxes over the image as appropriate. An additional lane box was drawn over an unloaded lane for use as a background control. Quantified areas (described in the figure legends) were manually designated with the band creation function. An additional, comparably sized “band” was designated in the blank lane to be used as the background control. The software was used to generate an analysis report containing the quantified band volume values. Background corrections were made by calculating the average signal per unit area in the background band box and subtracting the product of that value and the quantified area. Where applicable, *p*-values were obtained by performing an unpaired, two-tailed *t* test using GraphPad Prism 10 software.

## Data availability

All data that support this study are provided in the article.

## Supporting information

This article contains supporting information.

## Conflict of interest

The authors declare that they have no conflicts of interest with the contents of this article.
