# Supplementary material for: Defining a chromatin architecture that supports transcription at RNA polymerase II promoters
Source: J Biol Chem. 2024 Jun 28;300(8):107515. doi: 10.1016/j.jbc.2024.107515 (PMC11298586; doi:10.1016/j.jbc.2024.107515)
Supplement: Figure S1 [file mmc1.pdf]

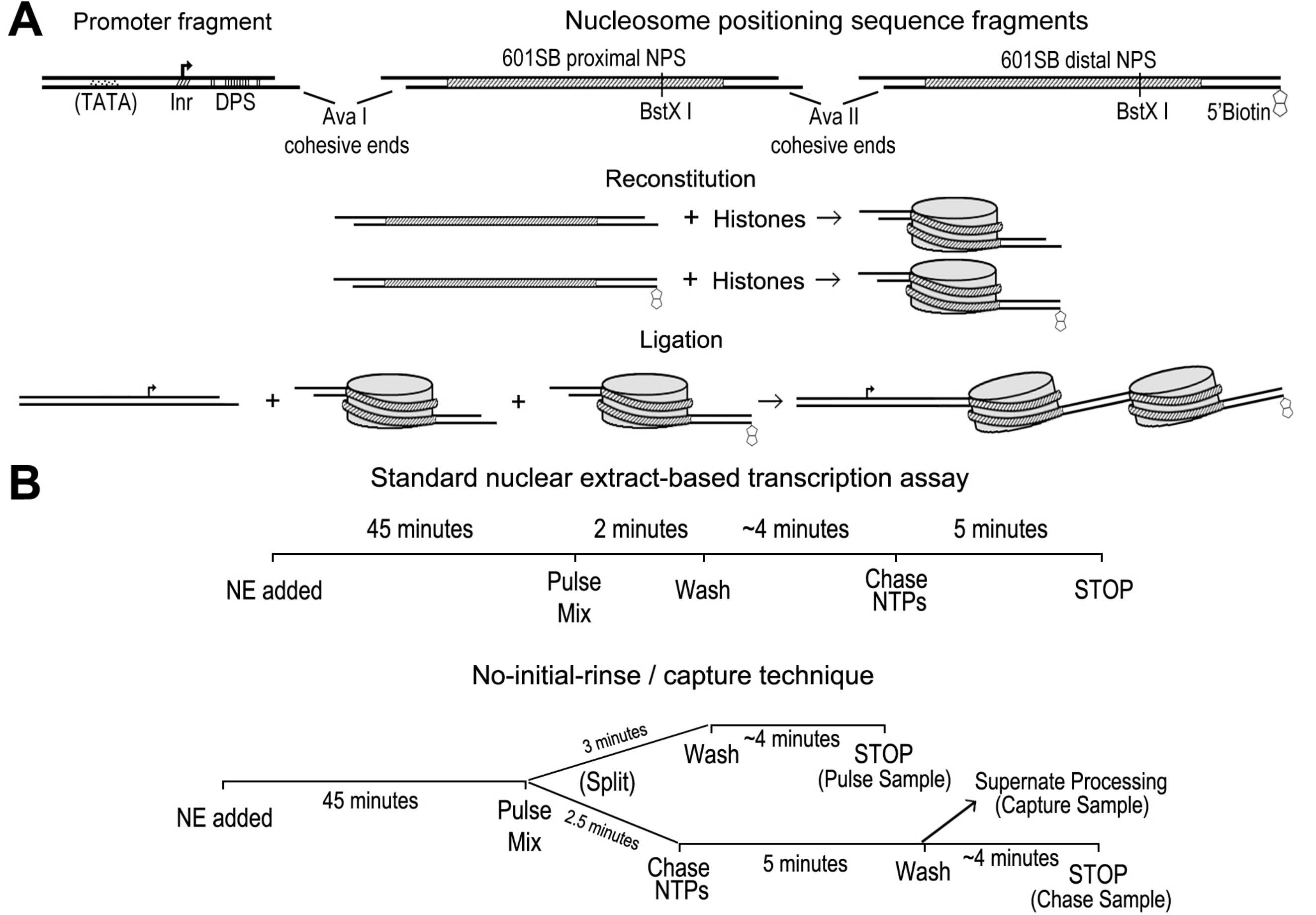

Fig. S1 Template assembly and in vitro transcription strategy. (a) DNA fragments containing nucleosome positioning sequences (NPS) and core promoters can be linked together through nonpalindromic cohesive ends prepared by Ava I or Ava II digestion. Nucleosomes may be reconstituted on NPS-containing fragments or left empty. NPS-containing fragments to be left empty lack the BstX I site so that a template completed as intended remains insensitive to BstX I digestion. (b) The standard nuclear extract-based in vitro transcription assay can be modified to retain nuclear extract factors during the chase and monitor released transcripts. Pulsed reactions are split into two equal volumes, only one of which is chased. After the 5 min chase reaction, the supernate is put into a separate tube and processed for oligonucleotide hybridization to capture the released transcripts. The pelleted beads are washed, incubated in STOP solution, then prepared for sample loading as normal.
