# Supplementary material for: Defining a chromatin architecture that supports transcription at RNA polymerase II promoters
Source: J Biol Chem. 2024 Jun 28;300(8):107515. doi: 10.1016/j.jbc.2024.107515 (PMC11298586; doi:10.1016/j.jbc.2024.107515)
Supplement: Figure S2 [file mmc2.pdf]

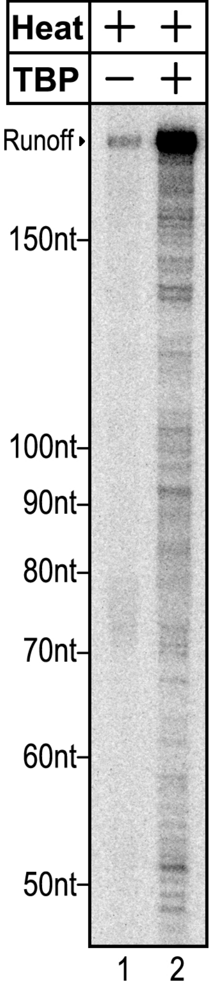

Fig. S2 TBP rescues transcription activity in heat-treated nuclear extracts. Transcription reactions using nuclear extract heat-treated for 15 minutes at 45 °C on HNRNPAB NPE 51 naked DNA templates (same as Figure 1) were supplemented with either BC100 (TBP buffer) or 10 ng of TBP.
