# Supplementary material for: Defining a chromatin architecture that supports transcription at RNA polymerase II promoters
Source: J Biol Chem. 2024 Jun 28;300(8):107515. doi: 10.1016/j.jbc.2024.107515 (PMC11298586; doi:10.1016/j.jbc.2024.107515)
Supplement: Figure S3 [file mmc3.pdf]

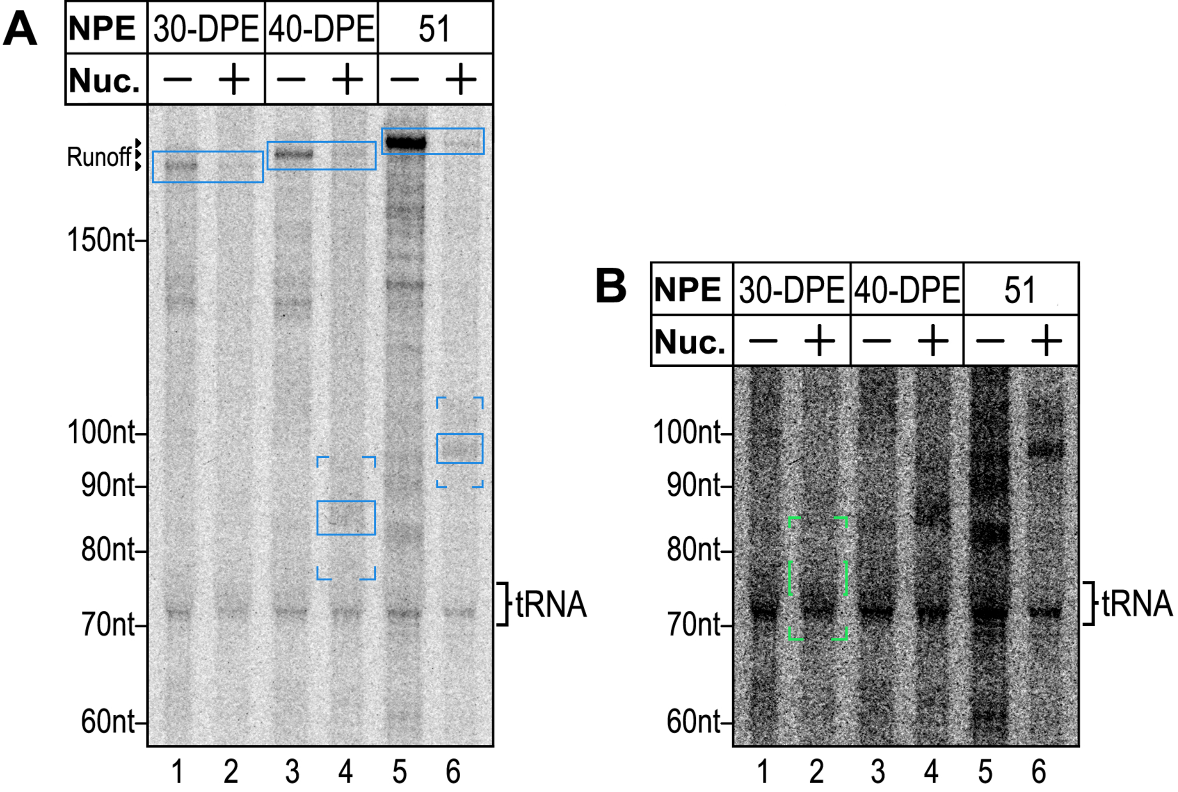

Fig. S3 Transcription of H3K4me3 nucleosome templates containing the KLHL15 core promoter with disrupted DPEs. (a) KLHL15 promoter templates with numerically indicated NPEs were transcribed +/- an H3K4me3 nucleosome. The downstream promoter elements (DPE) on these promoters were either left intact (51), partially disrupted (40-DPE), or completely disrupted (30-DPE). Sequences for each of these promoters are found in Table S1. Detectible nucleosome barriers are boxed and bracketed (Lanes 4 and 6). Runoffs are boxed at the top of the gel image. (b) A darker exposure of panel A emphasizes the conspicuous absence of any detectible nucleosome barrier band for 30-DPE (Lane 2) compared to 40-DPE and 51 (Lanes 4 and 6). The expected barrier location in lane 2 is marked with brackets.
