# Supplementary material for: Defining a chromatin architecture that supports transcription at RNA polymerase II promoters
Source: J Biol Chem. 2024 Jun 28;300(8):107515. doi: 10.1016/j.jbc.2024.107515 (PMC11298586; doi:10.1016/j.jbc.2024.107515)
Supplement: Figure S4 [file mmc4.pdf]

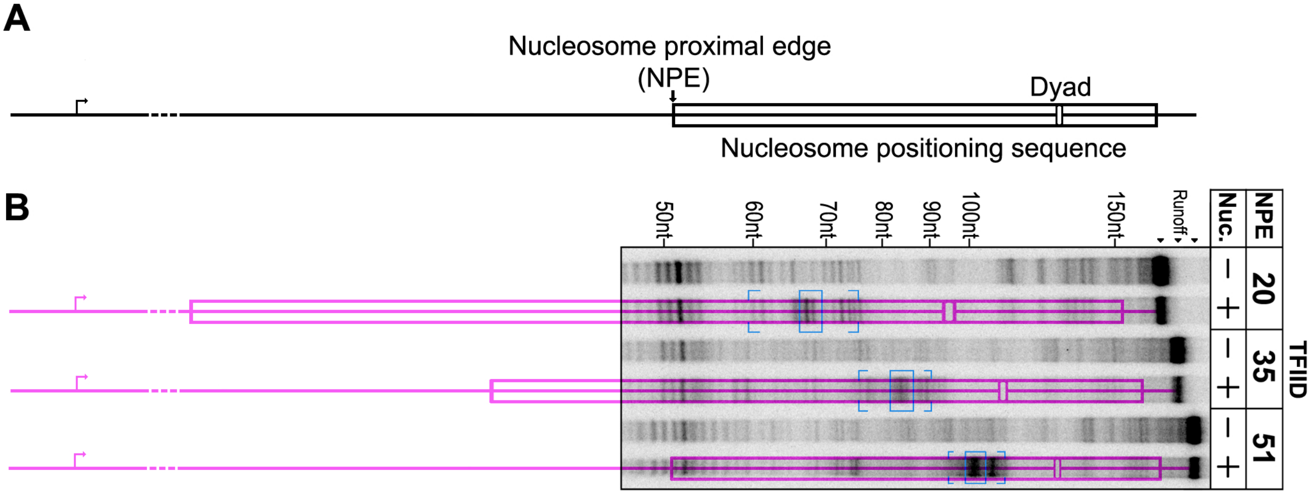

Fig. S4 Graphical comparison of sequencing gel outputs to templates. (a) Mononucleosome templates are represented graphically with a box indicating the 147 bp nucleosome positioning sequence, a white vertical bar indicating the dyad, and arrow representing the TSS. (b) The graphical representations of these templates, colored magenta, are overlayed on Figure 1 panel A (rotated 90 degrees clockwise) with the nucleosome positioning sequences adjusted to scale with the gel. The positions of the TSSs could not be rendered to scale and are arbitrarily designated, separated from the NPE with a dashed line.
