# Supplementary material for: Defining a chromatin architecture that supports transcription at RNA polymerase II promoters
Source: J Biol Chem. 2024 Jun 28;300(8):107515. doi: 10.1016/j.jbc.2024.107515 (PMC11298586; doi:10.1016/j.jbc.2024.107515)
Supplement: Table S1 [file mmc5.pdf]

A

601S	ACAGGATG**GACTTGA**CTGACACGTGCCTGGAGACTAGGGAGTAATCCCCTTGGCG<br>GTT**GTTAGCCTTGGATTG**GCGCGTACGTGCGTTTAAGCGGTGCTAGAGCTGTCTA<br>CGACCAATTGAGCGGCCTCGGCACCGGGATTCTCCAG
M13R	CAGGAAACAGCTATGACCATG
AML51_AvaI	TACTCCCGAGTTCCCAAAGGCCTTTAAAGAG
HNRNPAB-96	TACTAAGCTTGAGTGACGGAC
HNRNP51_AvaI	TACTCCCGAGACAGCCAAGCTCCGTC
HNRNP35_AvaI	TACTCCCGAGCCGCGCGGTGCC
HNRNP20_AvaI	TACTCCCGAGGCCTGACAATGCCGAC
M13F	TTGTAAAACGACGGCC
KLHL15-51_AvaI	TACTCCCGAGCGAGGAAGCGTCGAG
KLHL15-35_AvaI	TACTCCCGAGCTCTGGGCTTCTGCC
KLHL15-20_AvaI	TACTCCCGAGTTCTGCCCCGAAGCG
601KLHL15-20_AvaI	TACTCTCGGGAGACGCCGTCCTCGACGCTTCCTCGCTGACACGTGCCTGG
601KLHL15-35_AvaI	TACTCTCGGGTCCTCGACGCTTCCTCGACAGGATGGACTTGACTGAC
601R_AvaI	TACTCTCGGGCGCCAAGCTTACAGGATG
601S_Rev_AvaII	TACTGTGGTCCAATTCGAGCTCGGTATCCG
601S_Fwd_AvaII	TACTCTGGACCGCCAAGCTTACAGGATG
601R_Rev_Biot	5'-Biotin - AGTGAATTCGAGCTCGGTATCCGGGGATCCG
C_HNRNPAB20	AAGCTTGGCGCCCGAGGCCT 3' - Biotin
C_HNRNPAB51	TCCCGCGCGGTGCCGCCGCCT 3' - Biotin

B

Template_AML51	GACCATGATTACGCCAAGCTTAGCTTCCGGAAGGGGGGCTATAAAAGGGGGTGGGGGCGC<br>GCTCGTCCTCACTCTCTTCCCCTTCTCTTTAAAGGCCTTTGGGAA**CTCGGG**CGCCAAGCTT[A
Template_HNRNP20	GAGTGTGCAGTTTGTTGCGATGTGGCACCCGGCTCCGGCATTATAAAGGGCGCCACGAGT<br>CGGCATTGTCAGGC**CTCGGG**CGCCAAGCTT[ACAGGATGGACTTGACTGACACGTGCCTGGAG
Template_HNRNP35	GAGTGTGCAGTTTGTTGCGATGTGGCACCCGGCTCCGGCATTATAAAGGGCGCCACGAGT<br>CGGCATTGTCAGGCGGCGGCACCGCGCGG**CTCGGG**CGCCAAGCTT[ACAGGATGGACTTGACT
Template_HNRNP51	GAGTGTGCAGTTTGTTGCGATGTGGCACCCGGCTCCGGCATTATAAAGGGCGCCACGAGT<br>CGGCATTGTCAGGCGGCGGCACCGCGCGGGACGGAGCTTGGCTGT**CTCGGG**CGCCAAGCTT[A
Template_KLHL15-20	TTCCCCGCCCCTTTCCCGCCTTCCTCCACCCCCGGCGTGGGTGATCCGGAGGCTCGGCGC<br>GCTTCGGGGCAGAA**CTCGGG**AGACGCCGTC[CTCGACGCTTCCTCGCTGACACGTGCCTGGAG
Template_KLHL15-35	TTCCCCGCCCCTTTCCCGCCTTCCTCCACCCCCGGCGTGGGTGATCCGGAGGCTCGGCGC<br>GCTTCGGGGCAGAAGCCCAGAG**CTCGGG**TCCTCGACGCTTCCTCG[ACAGGATGGACTTGACT
Template_KLHL15-51	TTCCCCGCCCCTTTCCCGCCTTCCTCCACCCCCGGCGTGGGTGATCCGGAGGCTCGGCGC<br>GCTTCGGGGCAGAAGCCCAGAGACGCCGTCCTCGACGCTTCCTCG**CTCGGG**CGCCAAGCTT[A
KLHL15_30-DPE	GCTTCGGGGCAGAAGCCCAGAGAC**CTCGGG**CGCCAAGCTT[ACAGGATGGACTTGACTGACAC
KLHL15_40-DPE	GCTTCGGGGCAGAAGCCCAGAGACGCCGTCCTCG**CTCGGG**CGCCAAGCTT[ACAGGATGGACT

Table S1. DNA sequences for synthetic oligonucleotides, custom NPS, and promoters.
A: 601S is a modified 601 element nucleosome positioning sequence, the bold nucleotides are substituted from the corresponding sequence in clone 603. The underlined nucleotide is changed to a T in sequences containing a BstX I site. **B**: Sequences of promoters in complete templates from TSS -70 to +52 are also shown. TSS is underlined, Ava I site in bold, nucleosome proximal edge indicated with a bracket.
